# SAM protects against alveolar septal cell apoptosis in autoimmune emphysema rats

**DOI:** 10.1186/s40001-023-01396-w

**Published:** 2023-10-25

**Authors:** Dan Li, Ben-xue Li, Ye Zhang, Xia Li, Jia-yi Li, Xiang-yan Zhang, Xian-wei Ye, Cheng Zhang

**Affiliations:** 1 Department of Respiratory Medicine, Guizhou Provincial People’s Hospital, No. 83, Zhongshan East Road, Guiyang, Guizhou China; 2 NHC Key Laboratory of Pulmonary Immunological Diseases, Guizhou Provincial People’s Hospital, Guiyang, Guizhou China; 3 Panzhou People’s Hospital, Panzhou, Guizhou China

**Keywords:** *S*-Adenosylmethionine, Autoimmune emphysema, DNA methylation, Cell apoptosis, Rat

## Abstract

**Background:**

Hypomethylation of the perforin gene promoter in CD4 + T cells, inflammation and oxidative stress, might be involved in alveolar septal cell apoptosis associated with emphysema in rats. This study aimed to investigate the effects of *S*-adenosylmethionine (SAM) on this kind of apoptosis in rats with autoimmune emphysema.

**Methods:**

Twenty-four rats were randomly divided into three groups: a normal control group, a model group, and a SAM group. Pathological changes in lung tissues were observed, and the mean linear intercept (MLI) and mean alveolar number (MAN) were measured. The levels of anti-endothelial cell antibodies (AECA) in serum, alveolar septal cell apoptosis, perforin gene promotor methylation in CD4 + T cells in the spleen, and the levels of cytokines, malondialdehyde (MDA), and glutathione (GSH) and the activities of superoxide dismutase (SOD) and glutathione peroxidase (GSH-Px) in bronchoalveolar lavage fluid (BALF) were investigated.

**Results:**

The MLI, apoptosis index (AI) of alveolar septal cells, levels of AECA in serum, and levels of tumour necrosis factor-α (TNF-α), matrix metalloproteinase-9 (MMP-9) and MDA in BALF were increased, while the MAN, methylation levels, and the activities of GSH, SOD and GSH-Px in BALF were decreased in the model group compared with those in the normal control group and the SAM group (all *P* < 0.05). The levels of interleukin-8 (IL-8) in BALF were greater in the model group than in the normal control group (*P* < 0.05).

**Conclusions:**

SAM protects against alveolar septal cell apoptosis, airway inflammation and oxidative stress in rats with autoimmune emphysema possibly by partly reversing the hypomethylation of the perforin gene promoter in CD4 + T cells.

## Introduction

The pathology of chronic obstructive pulmonary disease (COPD) mainly includes obstructive bronchitis and pulmonary emphysema [1]. In addition to the three classic theories of the pulmonary inflammatory response, oxidative stress and protease-antiprotease imbalance, an imbalance in apoptosis and proliferation in alveolar septal cells (mainly alveolar epithelial cells and vascular endothelial cells) results in lung tissue damage associated with COPD. Therefore, some scholars consider this the fourth pathogenesis of COPD [2]. Smoking is a critical risk factor for this disease, but alveolar destruction associated with airspace enlargement typically progresses in severe COPD patients, although these individuals may have quit smoking many years ago [3]. Currently, it is accepted that the autoimmune response contributes to the progression of COPD, but the detailed mechanisms remain unclear.

Anti-endothelial cell antibodies (AECA) are circulating antibodies that bind to endothelial antigens and induce endothelial cell damage. Studies have shown that the AECA-mediated autoimmune response contributes to the development of alveolar septal cell apoptosis and COPD-associated emphysema [3–5] and that hypomethylation in the perforin gene promoter region is involved in some autoimmune diseases, such as systemic lupus erythematosus (SLE) [6] which was proven to be an important cause of COPD [7]. Whether hypomethylation is related to the autoimmune response in COPD-emphysema is not yet clear. Since the pulmonary inflammatory response and oxidative stress contribute to the development of COPD and our previous study showed that the methylation levels of the perforin gene promoter in CD4 + T lymphocytes were reduced and the AI of apoptotic alveolar septal cells was greater in rat models of autoimmune emphysema than in the normal group [8], we hypothesize that hypomethylation of the perforin gene promoter in CD4 + T lymphocytes are involved in alveolar septal cell apoptosis and that airway inflammation and oxidative stress play important roles in autoimmune emphysema in rats.

*S*-adenosylmethionine (SAM) is a methyl donor that is widely present in organisms and plays an important role in regulating gene expression. Some scholars have proven that it can reverse the hypomethylation of genes [9] and attenuate oxidative stress and inflammation [10]. Therefore, based on our previous study [8], we hypothesized that SAM treatment could prevent alveolar septal cell apoptosis by partly reversing the hypomethylation of this gene and attenuating airway inflammation and oxidative stress in rats with autoimmune emphysema. Whether this hypothesis is true remains to be determined.

In this study, we established a rat model of autoimmune emphysema and used SAM treatment to evaluate its effects on alveolar septal cell apoptosis associated with autoimmune emphysema in rats and shed light on a potential role for SAM as a novel therapeutic agent in this disease.

## Methods

### Cells

Umbilical cords were collected after the delivery of full-term normal pregnancies.

Human umbilical vein endothelial cells (HUVECs) were isolated and cultured within 6 h as described by Taraseviciene-Stewart et al. [4]. The experiments were approved by the Committee of Guizhou Provincial People’s Hospital.

### Animals

The experiments were approved by the Animal Ethics Committee of Guizhou Provincial People’s Hospital. Thirty-two male Sprague Dawley (SD) rats were purchased from Chongqing Tengxin Technology Co., Ltd. (weight ranging between 180 and 250 g, 10 weeks old). The rats were randomly divided into a SAM intervention group (*n* = 8), a model group (*n* = 8) and a normal control group (*n* = 8). The model group was intraperitoneally injected with HUVECs (1 × 10^7^) plus adjuvant once [4] and the same amount of normal saline as the SAM group every day. The SAM group was intraperitoneally injected with HUVECs (1 × 10^7^) plus adjuvant once and SAM (200 mg/kg per rat) dissolved in normal saline every day according to a reference [11] and our pre-experiment. SAM was provided by ABBOTT (Srl, Italy, registration certificate number for the imported drug: H20090409). The normal group was intraperitoneally injected with adjuvant once and the same amount of normal saline as the SAM group every day.

### Morphological assessment of lung tissue

The rats were intraperitoneally anaesthetized with 10% chloral hydrate (0.3 ml/100 g) on Day 21 of the experiment. The right lungs were filled with 4% paraformaldehyde for thirty minutes before being fixed in 4% paraformaldehyde for 24 h and then stained with haematoxylin–eosin (HE). Pathological changes were observed under a light microscope. The mean linear intercept (MLI) and mean alveolar number (MAN) of lung tissue were determined as described previously [12].

### Bronchoalveolar lavage and serum analysis

Bronchoalveolar lavage fluid (BALF) and serum were collected as described previously [12]. The levels of AECA in serum and matrix metalloproteinase-9 (MMP-9), tumour necrosis factor-α (TNF-α), AECA and interleukin-8 (IL-8) in BALF were determined using enzyme-linked immunosorbent assay (ELISA) according to the manufacturer’s protocol (BOSTER Company, USA). The levels of malondialdehyde (MDA), glutathione (GSH), superoxide dismutase (SOD) and glutathione peroxidase (GSH-PX) in BALF were detected using commercial kits (Nanjing Jiancheng Bioengineering Institute, Nanjing, China) according to the manufacturer’s instructions.

### Quantitative analysis of apoptosis

Alveolar septal cell apoptosis was detected by the terminal deoxynucleotidyl transferase-mediated dUTP nick end labelling (TUNEL) technique. The apoptosis index (AI) was calculated as described previously [12].

### CD4 + T-cell isolation, DNA extraction and next-generation sequencing

CD4 + T-cells were isolated from rat spleens, and DNA extraction and next-generation sequencing were performed to investigate perforin gene promotor methylation as described previously [8, 13]. The primers were designed for PCR amplification. The upstream primer was 5′-CTGCTAG AG AG GGT CTGAAGA-3′, and the downstream primer was 5′-TTCCCATGCT AGGCAAGT GGG ATAC-3′.

### Statistical analysis

SPSS 22.0 statistical software (IBM, USA) was used for data analysis. The data are expressed as the mean ± standard deviation. Significant differences were evaluated using analysis of variance. *P* values < 0*.*05 were considered to be statistically significant.

## Results

### Histological studies

**Fig. 1:**
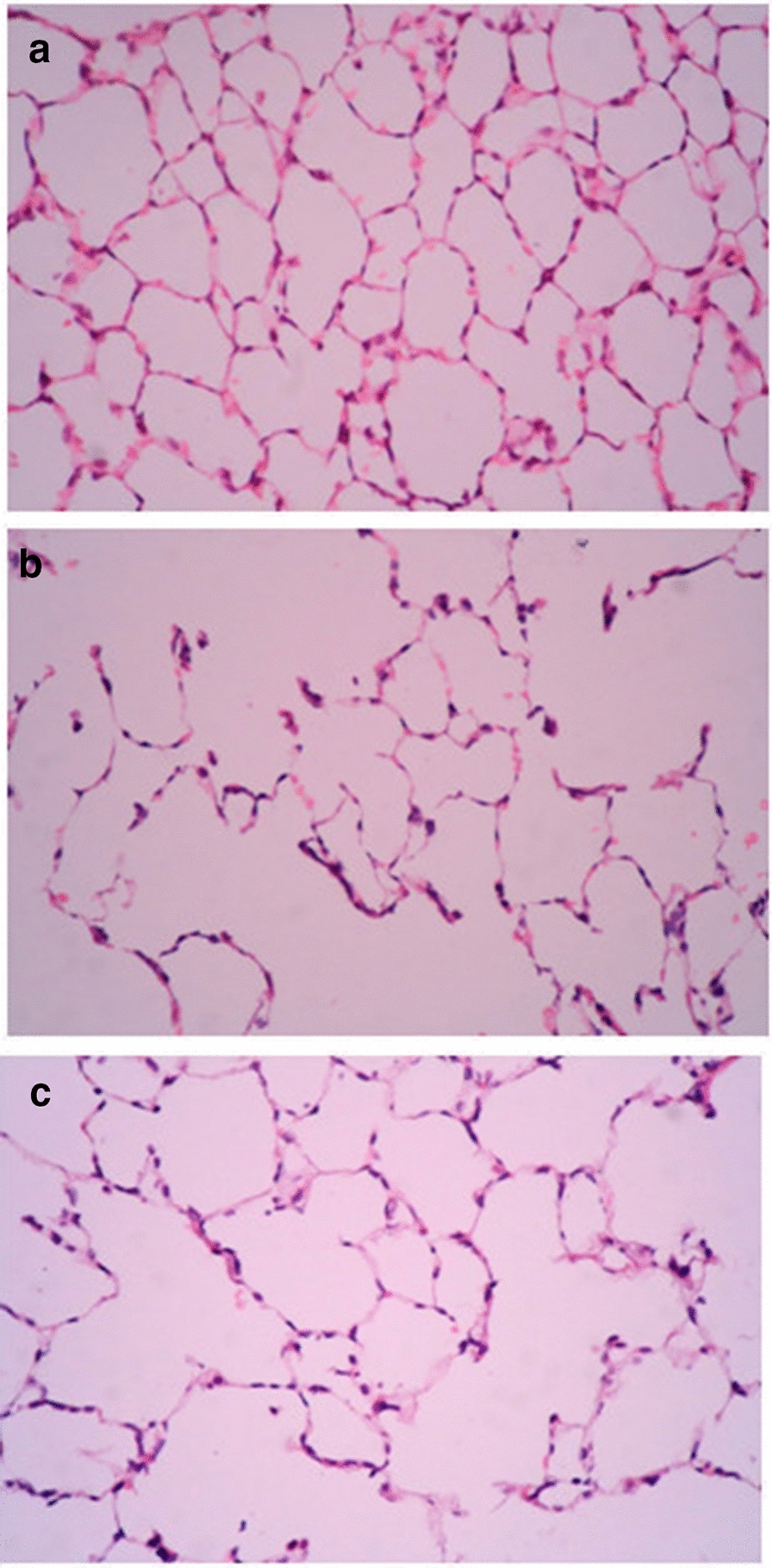
**a** Lung tissue histology in a normal control rat (HE, original magnification × 400). **b** Lung tissue histology in a rat with emphysema (HE, original magnification × 400). **c** Lung tissue histology in a rat treated with SAM (HE, original magnification × 400)

There were no pathological changes in pulmonary emphysema in the normal group (Fig. 1a), while in the lung tissue sections of the model group, parts of the pulmonary alveoli were enlarged and broken (Fig. 1b). Although pathological changes were observed in the SAM group (Fig. 1c), the degree of pulmonary emphysema was lower than that in the model group (Fig. 1b). The MLI was increased, while the MAN was decreased in the model group and the SAM group compared with the normal group. The MLI was decreased, while the MAN was increased in the SAM group compared with the model group. (All *P* < 0.05, Table 1).

**Table 1:** Differences in MLI and MAN in the three groups

Groups	*n*	MLI (× 10^–6^ m)	MAN (× 10^6^/m^2^)
Normal group	8	34.88 ± 3.44	673.00 ± 25.96
Model group	8	61.38 ± 3.46^a^	242.50 ± 39.04^a^
SAM group	8	46.38 ± 4.63^a,b^	426.88 ± 52.91^a,b^
*F* value		93.67	224.03

^a^*P* < 0.05, compared with the normal group

^b^*P* < 0.05, compared with the model group

### Levels of AECA, cytokines, MDA, GSH, SOD and GSH-PX

To investigate the role of AECA, cytokines and oxidative stress in autoimmune emphysema in rats and evaluate the effects of SAM, we detected the levels of AECA in serum and MMP-9, TNF-α, AECA, IL-8, MDA, GSH, SOD and GSH-PX in BALF. The MLI, AI of alveolar septal cells, levels of AECA in serum and levels of TNF-α, MMP-9 and MDA in BALF were increased, while the MAN, methylation levels, and activities of GSH, SOD and GSH-Px in BALF were decreased in the model group compared with the normal control group and the SAM group. The levels of IL-8 in BALF were greater in the model group than in the normal control group (all *P* < 0.05, Tables 2, 3).

**Table 2:** Differences in AECA levels in serum and TNF-α, IL-8 and MMP-9 levels in BALF in the three groups

Groups	*n*	AECA (ng/ml)	TNF-α (pg/ml)	IL-8 (pg/ml)	MMP-9 (ng/ml)
Normal group	8	14.56 ± 4.29	50.60 ± 10.94	8.99 ± 2.67	10.38 ± 1.92
Model group	8	55.04 ± 8.41^a^	90.38 ± 9.47^a^	11.72 ± 1.17^a^	18.04 ± 5.39^a^
SAM group	8	25.25 ± 5.24^a,b^	57.42 ± 11.61^b^	11.63 ± 1.09^a^	12.78 ± 2.88^b^
*F* value		90.59	31.56	5.98	9.05

^a^*P* < 0.05, compared with the normal group

^b^*P* < 0.05, compared with the model group

**Table 3:** Differences in MDA, GSH, SOD and GSH-Px levels in BALF in the three groups

Groups	*n*	MDA (nmol/ml)	GSH (μmol/L)	SOD (U/ml)	GSH-Px (U/ml)
Normal group	8	28.54 ± 9.04	0.78 ± 0.12	100.65 ± 8.59	18.11 ± 3.22
Model group	8	253.43 ± 41.80^a^	0.23 ± 0.11^a^	64.90 ± 4.59^a^	6.11 ± 1.93^a^
SAM group	8	140.52 ± 17.36^a,b^	0.54 ± 0.14^a,b^	84.98 ± 4.73^a,b^	13.52 ± 1.11^a,b^
*F* value		142.49	39.72	65.77	57.44

^a^*P* < 0.05, compared with the normal group

^b^*P* < 0.05, compared with the model group

### Quantitative analysis of apoptosis in the alveolar septum

**Fig. 2:**
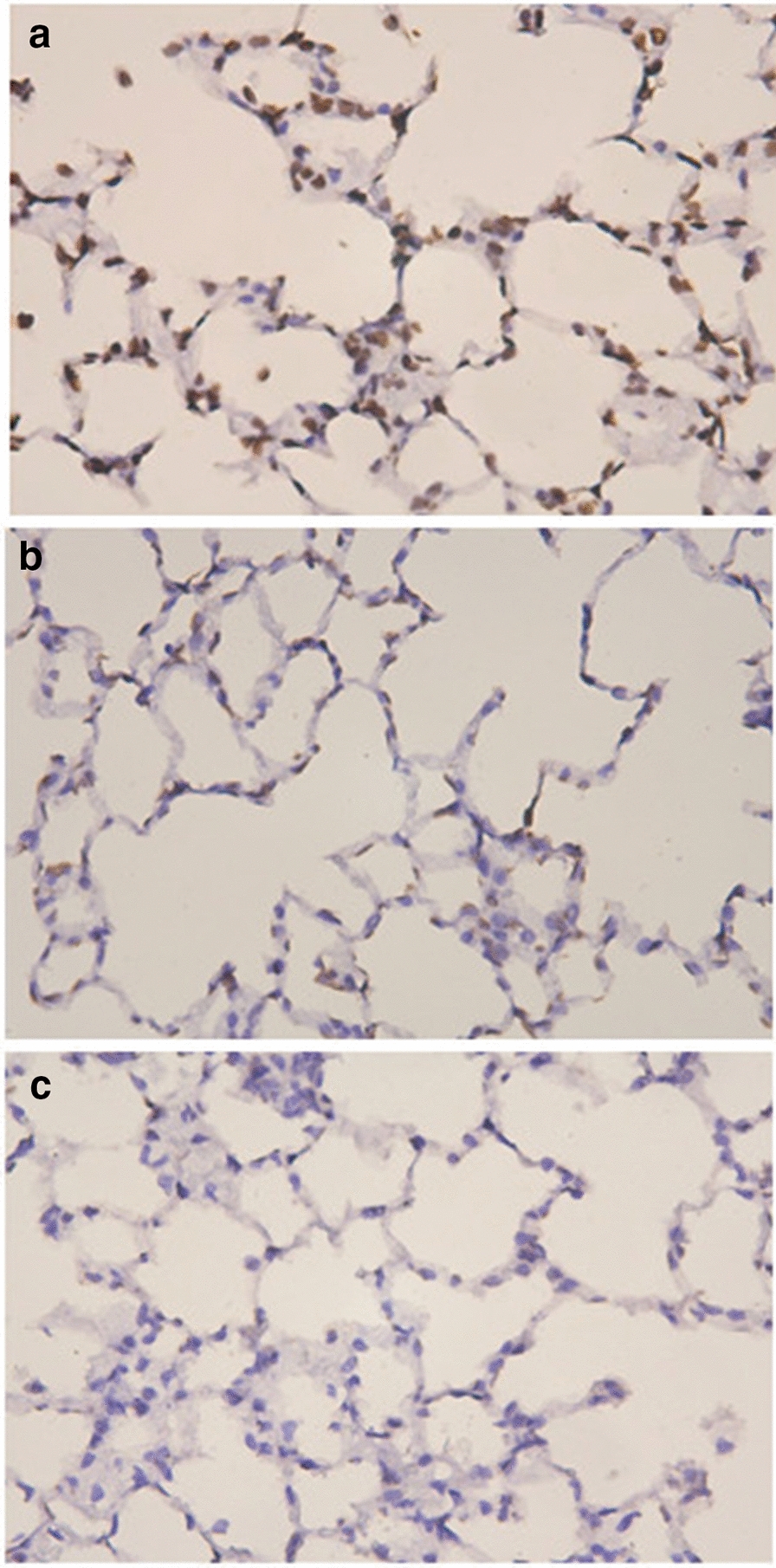
**a** Alveolar septal cell apoptosis in a rat with emphysema (TUNEL, original magnification × 400). **b** Alveolar septal cell apoptosis in a rat receiving SAM (TUNEL, original magnification × 400). **c** Alveolar septal cell apoptosis in a normal control rat (TUNEL, original magnification × 400)

Alveolar septal cell apoptosis was detected in the lung tissues of the three groups. TUNEL-positive cells were mainly localized in the alveolar septa. In the model group, there was an increase in apoptotic cells in the alveolar septum (Fig. 2a). The AI was higher in the model group (Fig. 2a) and the SAM group (Fig. 2b) than in the normal group (Fig. 2c), and it was lower in the SAM group (Fig. 2b) than in the model group (Fig. 2a) (All *P* < 0.05, Table 4).

**Table 4:** Difference in the AI of alveolar septal cells in the three groups

Groups	*n*	AI (%)
Normal group	8	9.00 ± 2.51
Model group	8	27.00 ± 3.70^a^
SAM group	8	15.63 ± 3.93^a,b^
*F* value		28.3

^a^*P* < 0.05, compared with the normal group

^b^*P* < 0.05, compared with the model group

### *Methylation levels of the perforin gene promoter in CD4* + *T cells*

Methylation (− 1376 to − 1200 bp) of the perforin gene promoter in the CD4 + T lymphocytes of rats is shown in Table 5. The methylation levels in the model group were lower than those in the normal group (*P* < 0*.*05). The levels were higher in the SAM group than in the model group (*P* < 0*.*05, Table 5).

**Table 5:** Levels of perforin gene promoter methylation in CD4 + T cells

Groups	*n*	Methylation levels (× 10^–2^)
Normal group	8	93.14 ± 0.55
Model group	8	91.68 ± 1.25^a^
SAM group	8	93.19 ± 0.74^b^
*F* value		7.32

^a^*P* < 0.05, compared with the normal group

^b^*P* < 0.05, compared with the model group

## Discussion

Inflammation in COPD patients remains persistent and progressive even if the patients have stopped smoking. Some related studies have suggested that the autoimmune response may be involved in the development of COPD-associated emphysema [3–5], but the mechanisms are not yet clear and need further study.

In this study, we injected rats with xenogeneic endothelial cells to establish an autoimmune emphysema model. We found that pathological changes associated with pulmonary emphysema occurred in the model group. Compared with those in the normal group, the MLI increased while the MAN decreased. Furthermore, the levels of AECA in serum and TNF-α, MMP-9 and IL-8 in BALF were significantly higher than those in the normal group, which was similar to the characteristics of autoimmune models reported by Taraseviciene-Stewart et al. [4], indicating that the autoimmune emphysema model in this study had been successfully established.

Apoptosis, which is a type of cell death, is controlled by the interactions between several molecules and is responsible for the elimination of unwanted cells from the body [14]. Abnormal apoptosis is closely related to various diseases, such as emphysema. Some scholars have shown that AECA, airway inflammation and oxidative stress are involved in the pathogenesis of COPD-associated emphysema [4, 5, 15, 16]. In this study, we found that the AI of alveolar septal cells, the levels of AECA in serum and the levels of MDA, MMP-9, TNF-α and IL-8 in BALF were increased, while the activities of GSH, SOD and GSH-Px in BALF were decreased in the model group compared with the normal control group, suggesting that airway inflammation and oxidative stress play an important role in autoimmune emphysema in rats, which is similar to our previous study [8].

DNA methylation is an epigenetic modification involving the transfer of a methyl group to the C5 position of cytosine to form 5-methylcytosine. This modification regulates gene expression by recruiting proteins involved in gene repression or by inhibiting the binding of transcription factors to DNA. Taraseviciene-Stewart et al. [4] showed that the adoptive transfer of pathogenic, spleen-derived CD4 + T cells into naive immunocompetent rats resulted in emphysema. We hypothesize that CD4 + T-cell-dependent mechanisms may trigger the development of alveolar septal cell apoptosis and experimental emphysema. This study showed that the methylation levels of the perforin gene promoter in CD4 + T lymphocytes were lower in rats with autoimmune emphysema than in rats in the normal group. This finding was consistent with what had been observed in previously established autoimmune emphysema models [8], further suggesting that hypomethylation of this gene region is critical in alveolar septal cell apoptosis and the pathogenesis of autoimmune emphysema in rats. Therefore, we hypothesize that partial reversal of hypomethylation may attenuate cell apoptosis and this kind of disease in rats.

SAM is a physiologically active substance involved in cell metabolism in all tissues and fluids in the human body, and it is an important methyl donor related to normal cell function and survival. In addition, SAM is a potent antioxidant and can inhibit the autoimmune response [17, 18]. In one study, SAM was used to intervene in chronic asthma models, and it was found that airway infiltration of inflammatory cells was significantly decreased in the SAM-treated group [19]. Zhao et al. [9] showed that SAM inhibited the growth of human gastric cancer cells in vivo and in vitro by reversing the hypomethylation of certain related genes to suppress their overexpression, demonstrating that SAM could increase the levels of DNA methylation and prevent the progression of diseases, which provides a theoretical basis for exploring the effects of SAM on autoimmune emphysema in rats.

In this study, we found that MLI and the levels of AECA, MDA, MMP-9, TNF-α and AI in alveolar septal cells were decreased, while MAN, the activities of GSH, SOD and GSH-Px in BALF and methylation levels were increased in the SAM group compared with the model group. Our study revealed that SAM treatment protected against alveolar septal cell apoptosis, airway inflammation and oxidative stress in rats with autoimmune emphysema, possibly by partially reversing hypomethylation of the perforin gene promoter in CD4 + T cells. However, the specific mechanism of hypomethylation in this disease is not yet clear and remains to be further studied.

Some related studies have confirmed the important role of the perforin pathway in the induction of apoptosis and autoimmune disorders [20–26], which revealed the importance of perforin in immune-mediated diseases. This conclusion is also consistent with the result that the AI of alveolar septal cells was greater in the model group than in the normal control group. It is widely accepted that DNA hypermethylation inhibits the activity of related genes. Based on our previous study [8] and this study, we hypothesized that the perforin gene promoter in CD4 + T lymphocytes in rats with autoimmune emphysema was hypomethylated, which could activate perforin genes to promote the production of AECA and increase alveolar septal cell apoptosis, thereby contributing to the development of autoimmune emphysema in rats.

Therefore, we conclude that SAM protects against alveolar septal cell apoptosis, airway inflammation and oxidative stress in rats with autoimmune emphysema by partly reversing the hypomethylation of the perforin gene promoter in CD4 + T cells.

## Data Availability

The data are available from the corresponding author upon reasonable request.

## Abbreviations

SAM: *S*-Adenosylmethionine
MLI: Mean liner intercept
MAN: Mean alveolar number
AECA: Anti-endothelial cell antibodies
MDA: Malondialdehyde
GSH: Glutathione
SOD: Superoxide dismutase
GSH-Px: Glutathione peroxidase
BALF: Bronchoalveolar lavage fluid
AI: Apoptosis index
TNF-α: Tumour necrosis factor-α
MMP-9: Matrix metalloproteinase-9
IL-8: Interleukin-8
COPD: Chronic obstructive pulmonary disease
SLE: Systemic lupus erythematosus
HUVECs: Human umbilical vein endothelial cells
SD: Sprague Dawley
HE: Haematoxylin–eosin
TUNEL: Terminal deoxynucleotidyl transferase-mediated dUTP nick end labelling
